# 

**Published:** 1879-10-20

**Authors:** 


☞ TO SUBSCRIBERS IN ARREARS.—We are very thankful to those who have recently remitted their subscriptions; they are but few. We trust all others who have not done so will at once give attention to the matter.

### *EDITORIAL NOTICES.*

*Drugs in Atlanta.*—The drug market in Atlanta is well supplied, there being a number of large wholesale houses who are selling drugs on favorable terms. Among these houses we take pleasure in recommending as safe and reliable, the firm of Pemberton, Samuels & Reynolds. See their advertisement in this journal.

*John C. Baker & Co.*, Philadelphia.—Be sure and read their advertisement of COD LIVER OIL, with EXTRACT OF MALT, which we have reasons to know is an excellent preparation. This house, we are assured, stands deservedly high, and their preparations generally are reliable and excellent.

*Surgical Instruments, Saddle-Bags, Electric Apparatus, etc.*—The attention of medical students and physicians is specially directed to the House of Aloe & Hernstein, St. Louis, Mo. See their new advertisement, commencing with this issue of our journal. We recommend with much pleasure this reliable and excellent House. Their goods may be ordered from them direct, or obtained through the agency of any drug house.

In Atlanta, Pemberton, Samuels & Reynolds keep their saddle-bags, and will supply on short notice other goods from the St. Louis House.

*Beautiful Drug Samples.*—We have been kindly furnished with beautiful samples in the drug line from that staunch and popular House McKESSON & ROBBINS, New York. These we will place on deposit in the Medical College Dispensary, where they can be well displayed and also utilized—and where the liberality and enterprise of the donors will meet with just appreciation.

Other drug houses have also promised similar contributions, which after their arrival will be duly mentioned.

### *AMERICAN PUBLIC HEALTH ASSOCIATION.*

The above Association is appointed to meet in Nashville on the 18th November next.

The Sanitary Council of the Mississippi Valley and the Tennessee Medical Society will also meet at the same time and place.

A circular containing the programme and topics for discussion will soon be distributed by J. B. Lindsley, the Secretary.

The occasion is looked forward to as one of great interest to the Profession and to the public.

### *PAMPHLETS RECEIVED.*

FIRST STEP IN CHEMICAL PRINCIPLES, an introduction to modern chemistry, intended especially for beginners: By Henry Leffman, M. D., Lecturer on Texicology in the Summer School of Jefferson Medical College; Assistant Professor of Chemistry in Philadelphia Central High School; Lecturer on Chemistry at the Wagner Free Institute of Science, etc., etc. Price 50 cents.

A compact and admirable little work in which is condensed a vast amount of information in a small space. It will prove a valuable acquisition, not only "to beginners," but to advanced students of chemical science.

ARCHIVES OF MEDICINE, a Bi-Monthly Journal, edited by E. C. Seguin, M. D. Assistant editors: Thomas A. McBride, M. D., Matthew D. Mann, M. D., and Lewis A. Stimson, M. D. New York: G. P. Putnam's Sons, 182 Fifth Avenue. 1879.

URETHRISMUS, OR CHRONIC SPASMODIC STRICTURE: By F. N. Otis, M. D., clinical Professor of genito-urinary diseases in the College of Physicians and Surgeons, New York, etc.

PROCEEDINGS OF THE ALUMNI ASSOCIATION OF RUSH MEDICAL COLLEGE, Chicago. 1879.

DIPHTHERIA, ITS HISTORY, SYMPTOMS, CAUSES, PREVENTION AND CURE. Scarlet fever and its successful treatment. By Melville G. Keith, M. D. Lincoln, Nebraska: S. L. Moser, publisher. 1879.

REPORT ON ACONITIA IN TRIGEMINAL NEURALGIA: By E. C. S. Seguin, M. D. Reprinted from the New York Medical Journal, December, 1878. New York: D. Appleton & Co., 549 and 551 Broadway. 1878.

NORMAL POSITION AND MOVEMENTS OF THE UNIMPREGNATED UTERUS: By Ely Van de Warker, M. D., Syracuse, N. Y., Fellow of the American Gynecological Society. New York: William Wood & Co., 27 Great Jones street. 1878.

LACERATION OF THE CERVIX UTERI: By A. Reeves Jackson, A. M., M. D., formerly Surgeon-in-Chief of the Woman's Hospital of the State of Illinois; late Lecturer on the surgical diseases of women; in Rush Medical College; Fellow of the American Gynecological Society, etc., etc. Read before the Chicago Medical Society, July 7th, 1879. Reprinted from the Chicago Medical Journal and Examiner, for August, 1879.

LARYNGEAL TUMORS AND TUBERCULOUS LARYNGITIS: By E. Fletcher Ingals, A. M., M. D.; Lecturer on diseases of the chest and physical diagnosis and on laryngotomy, in the Post Graduate Course, Rush Medical College. Reprinted from the Chicago Medical Journal and Examiner, for July, 1879.

THE RADICAL CURE OF HERNIA BY THE ANTISEPTIC USE OF THE CARBOLIZED CATGUT LIGATURE: By Henry O. Marcy, A. M., M. D., Cambridge, Mass ; member of the Massachusetts Medical Society, American Medical Association; Cambridge Medical Improvement Society; late Surgeon United States Army, etc.

THE TREATMENT OF EPITHELIOMA OF THE CERVIX UTERI: By J. Marion Sims, M. D.; Founder of the Woman's Hospital of the State of New York, and formerly Surgeon to the same; ex-President of the American Medical Association, etc., etc.

A CLINICAL TREATISE ON DISEASES OF THE NERVOUS SYSTEM: By M. Rosenthal, Professor of diseases of the nervous system, with a preface by Professor Charcot. Translated from the author's revised and enlarged edition by L. Putzel, M. D., visiting Physician for nervous diseases Randall's Island Hospital; Physician to the class for nervous diseases, Bellevue Hospital out door department., etc., etc.—Vol. II., New York: Wm. Wood & Co., 27 Great Jones street. 1879.

This is the second volume of this valuable treatise, the first of which we noticed in a previous number of this journal. It is an admirable work, replete with instruction and interest on a department of which less is known than, perhaps, any other branch of medical science. It is 277 octavo pages, neatly gotton up and illustrated.

---

### *OBITUARY.*

DIED, in Hopkinsville, Ky,, September 30th, 1879, Rev. Gideon B. Perry, D.D., L.L.D.

Dr. Perry was an eminent divine, and received also in addition to the degrees named, that of M.D., in Jefferson Medical College, Philadelphia. Truly a great and good man is gone!

---

RECEIPTED.—1879: L. G. Hardman; J. J. Grace; E. N. Cushing; J. T. Sego; J. W. Huff; A. F. Sanders; J. R. Wilson; A. J. Eilis; Gillispie & Payne; S. L. Lockwood; G. B. Battle'; A. L. Barry; A. J. Sewell; W. S. Posey; R. J. McMullen; J. W. Bennett; Geo. L. Mills; J. W. Buddeke.

